# Preliminary Efficacy/Feasibility Study of a Breast Cancer-Related Lymphedema Prospective Screening and Early Intervention Program at the Dana-Farber Brigham Cancer Center

**DOI:** 10.3390/jcm14197051

**Published:** 2025-10-06

**Authors:** Sara P. Myers, Jacob M. Jasper, Tessa Higgins, Angela Serig, Amanda C. Faust, Lila J. Tappan, Faina Nakhlis, Erin M. Taylor, Shailesh Agarwal, Elizabeth A. Mittendorf, Tari A. King

**Affiliations:** 1 Division of Breast Surgery, Department of Surgery, Brigham and Women’s Hospital, Boston, MA 02115, USA; jacob.jasper@tufts.edu (J.M.J.);; 2 Breast Oncology Program, Dana-Farber Brigham Cancer Center, Boston, MA 02115, USA; 3 Department of Surgery, Harvard Medical School, Boston, MA 02115, USA; 4 Department of Surgery, Tufts University School of Medicine, Boston, MA 02111, USA; 5 Department of Rehabilitation Services, Brigham and Women’s Faulkner Hospital, Boston, MA 02130, USA; 6 Division of Plastic and Reconstructive Surgery, Department of Surgery, Brigham and Women’s Hospital, Boston, MA 02115, USA

**Keywords:** breast cancer, lymphedema, clinical protocol, patient-reported outcome measures, quality of life

## Abstract

**Background**: Breast cancer-related lymphedema (BCRL) is a common and debilitating treatment-related adverse event that can profoundly impact quality of life and financial well-being. Although prospective surveillance and early intervention for BCRL have been shown to reduce the incidence and severity of this chronic condition, diagnostic accuracy of screening, programmatic utilization and efficacy vary widely. We describe the protocol for the BCRL Prospective Surveillance Model (PSM) and Early Intervention Program at the Dana-Farber Brigham Cancer Center that aims to address these issues by augmenting arm measurements (standard of care) with use of patient-reported outcome metrics (PROMs). **Methods**: Women with newly diagnosed stage I-III breast cancer at high risk for developing BCRL based on tumor and treatment characteristics are eligible for inclusion in our PSM care pathway, which uses both the Breast Cancer and Lymphedema Symptom Experience Index PROMs and arm measurements for screening. Screening begins prior to the initiation of neoadjuvant therapy and continues at regular intervals postoperatively. A positive screen, defined as new patient-reported arm swelling/heaviness and/or relative volume change (RVC) ≥ 5% in the affected limb, triggers consideration for multidisciplinary early intervention. **Analysis**: The BCRL detection rate will be compared to years previous to protocol development. PSM feasibility will be determined according to the Reach, Effectiveness, Adoption, Implementation, and Maintenance (RE-AIM) framework. Efficacy of the PSM will be gauged by comparing change in patient-reported outcomes of interest and arm volume measurement pre and post early intervention. Feasibility will be determined by calculating the percentage of PSM-eligible individuals who complete all PSM activities in a 1-year span. Characteristics of participants versus non-participants in the target population will be compared. Furthermore, 1:1 semi-structured interviews with enrolled patients will be performed to understand facilitators and barriers to implementation. **Conclusions**: The findings from this study will be used to develop a standardized approach to PSM and early intervention that can be adapted to both resource-modest and resource-abundant healthcare infrastructures.

## 1. Introduction

Lymphedema is one of the most common treatment-related adverse events experienced by breast cancer survivors [1]. Up to 30% [2] of the 4.1 million breast cancer survivors in the United States will develop subclinical or clinical breast cancer-related lymphedema (BCRL). Reducing the incidence and impact of this ailment is critical to preserving quality of life during survivorship [3,4]. Although those who develop chronic BCRL require lifelong treatment, emerging evidence highlights the opportunity to intervene during the early phase of disease, at which point accumulation of extracellular fluid is reversible [5,6]. Accordingly, clinical guidelines emphasize the importance of prospective surveillance and early intervention programs in preventing chronic BCRL [7,8,9].

Despite the widely recognized importance of screening for BCRL, there are numerous challenges that impede efficacy, implementation and sustainability in routine clinical practice [10]. Groundbreaking work by Koelmeyer and colleagues [10] identified that the organizational readiness and programmatic efficacy of a prospective surveillance model (PSM) for BCRL rehabilitation were predicated on (1) sufficient provider and patient education, (2) process recognition and approval by staff and (3) delineating a formal referral and care pathway. Even for institutions that have established well-integrated PSMs, underdiagnosis of BCRL is a major barrier to referral for treatment and early intervention because severity of perceived symptoms may not correspond to degree of objective changes in arm volume [11].

Therefore, further work is needed to develop a PSM that incorporates objective assessment for diagnosis of BCRL, symptom evaluation for early and subclinical BCRL, and ongoing evaluation to ensure successful process implementation. In this manuscript, we describe the Dana-Farber Brigham Cancer Center’s (DFBCC) experience creating a PSM that addresses these issues. We aimed to develop a standardized approach to screening that assists providers in accurately identifying subclinical and clinical BCRL, could be easily replicated in both resource-plenty and resource-modest settings, and will mitigate downstream BCRL-associated sequelae for patients. By comparing data from this protocol to patient outcomes prior to its development, we will be able to assess whether patient-reported outcome metrics (PROMs) improve the diagnostic accuracy over use of arm measurement with bioimpedance testing alone (standard of care), and consequently, improve efficacy of early intervention.

## 2. Methods and Approach to Developing the PSM

### 2.1. PSM Personnel and Patient Selection

The complexity of BCRL necessitates a multidisciplinary approach to screening, diagnosis and treatment [12]. Given the frequency at which BCRL develops among breast cancer survivors, we advocate that providers should engage in discussions about screening from the outset of treatment planning. At our institution, breast surgical oncologists spearhead efforts for BCRL surveillance and early intervention. The members of the surgical team are well positioned for this role because they are among the first providers to meet patients after a new breast cancer diagnosis and because operative management of the axilla heightens risk for developing BCRL.

In addition to breast surgical oncology champions (T.A.K., E.A.M. and S.P.M.), a PSM requires 3–4 staff members (depending on patient volume) to educate patients, perform arm measurements and monitor data collection. In our program, a certified lymphedema therapist (AS) oversees these activities and facilitates referral for early intervention. To improve upon the quality of the clinical services provided, this team also cooperates on academic initiatives related to the PSM, early intervention and BCRL treatment.

Identifying the patients who would most benefit from surveillance and early intervention for BCRL is critical to designing and implementing a feasible and effective PSM [13]. Healthcare structures, especially in resource limited settings, may not be prepared to accommodate the cost or workforce requirements of a PSM that aims to screen all patients [14]. To address this, we propose an individualized approach that targets screening to those at highest risk for BCRL. The literature has identified that patients may be at high risk for developing BCRL based on baseline and treatment characteristics including BMI at time of diagnosis [15], tumor size [16], receipt of taxane-based chemotherapy [17], number of lymph nodes removed and/or axillary lymph node dissection (ALND) [18,19], and regional nodal irradiation [20]. Accordingly, in an attempt to enrich the population with those at greatest risk for developing BCRL who would have most to benefit, patients receiving neoadjuvant therapy (NAT) with or without clinically node-positive disease and/or patients for whom ALND is planned are eligible for enrollment in our PSM. These criteria were selected by a multidisciplinary team of breast oncology experts using a modified Delphi consensus approach [21] that allowed for initial input, synthesis and feedback, iterative refinement and consensus on criteria by majority agreement.

To identify eligible patients, members of the lymphedema screening team review surgeons’ scheduled new consult visits for relevant data including diagnostic imaging and pathology to determine whether a patient is likely to receive NAT and therefore meet inclusion criteria (Table 1). After confirming that the patient meets eligibility criteria and that the patient is interested in participating, patients have their initial screening visit. Although patients who undergo surgical treatment for their breast cancer at non-DFBCC sites are excluded, receipt of NAT or adjuvant radiation at facilities external to DFBCC does not preclude participation in the PSM. The lymphedema team also reviews established consults to verify whether there are patients who have completed NAT, have not yet had surgery and may have been overlooked during the pre-NAT visit with their surgeon.

**Figure 1:**
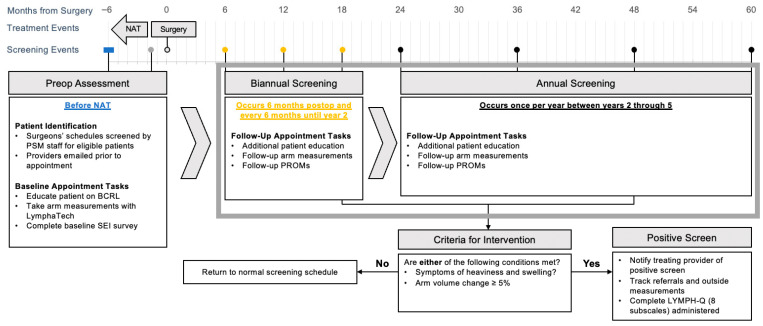
Dana-Farber Brigham Cancer Center prospective BCRL surveillance model. Abbreviations: NAT, neoadjuvant therapy; PSM, prospective surveillance model; BCRL, breast cancer-related lymphedema; SEI, Symptom Experience Index; PROM, patient-reported outcome metric.

Our PSM workflow (Figure 1) is designed to assess the impact of screening and early interventions on clinical outcomes and PROs over time. Specifically, data for enrolled patients are recorded and stored in the institutional REDCap database and linked to their electronic medical record. The workflow schema is organized into three phases based on timing of surgery.

### 2.2. Initial Intake (Baseline) Appointment

Ideally, a preoperative assessment is performed prior to NAT initiation. For patients who are unable to complete their preoperative assessment before commencing NAT, baseline assessments are recorded after completion of NAT but prior to surgery. This distinction is documented and necessary for future analyses given concern that chemotherapy may result in upper extremity symptoms [22] or increase risk for BCRL (although the literature regarding the latter is inconsistent) [23].

At the baseline appointment, the patient is educated regarding lymphedema including risk factors, symptoms, risk-reduction strategies, and treatment. The goals of the screening program are discussed, and the patient is instructed to notify their clinical team if they experience lymphedema-related symptoms, specifically swelling or heaviness in the arm, or if clothes or jewelry begin to feel tight on the affected side. Baseline limb volume measurements are then obtained using the LymphaTech device. Within 24 h following the baseline appointment, an invitation to complete the BCLE-SEI and LYMPH-Q ‘information’ subscale are sent to the patient via REDCap 15.0.34.

### 2.3. Bi-Annual Screening: 6–24 Months Postoperatively

BCRL onset varies with treatment strategy; however, for those receiving ALND alone, BCRL may begin to develop as early as 6 months postoperatively [9]. Accordingly, the DFBCC screening protocol seeks to schedule the first postoperative visit within this 6-month time frame. Because this PSM appointment aligns with routine medical and surgical oncology appointments at our institution, the visit is convenient for patients and allows for multidisciplinary care coordination.

### 2.4. Annual Screening After 24 Months Postoperatively

To minimize strain on institutional resources and accommodate patient capacity, our protocol transitions patients out of semi-annual screening into annual screening 24 months after surgery. This procedure was established based on a prospective study of 2171 women conducted at Massachusetts General Hospital that demonstrated, for the majority of treatment regimens, BCRL incidence declines after 24 months [24]. Screening at annual rather than semi-annual intervals continues until 5 years postoperatively as most patients who develop BCRL do so within this time period [25].

### 2.5. Metrics Used in Screening

**Figure 2:**
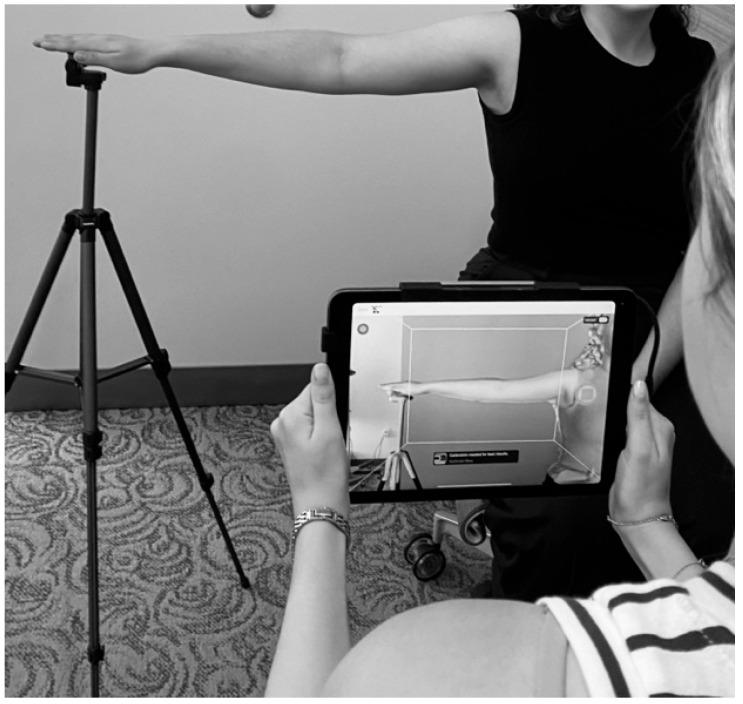
LymphaTech imaging system measurement.

While we recognize multiple modalities are available for BCRL diagnosis, our program employs the LymphaTech 3D Imaging System (LymphaTech, Atlanta, GA, USA) to detect changes in upper extremity volume. The LymphaTech system has several advantages: it is accurate, fast, portable across multiple clinic rooms and sites [26], and has demonstrated validity and reliability [27]. Volume measurements are stored in an encrypted device within the LymphaTech application and a unique identifier is assigned for each patient. Measurements are obtained with the patient seated upright in a chair with the arm abducted 90 degrees. The lymphedema team member instructs the patient on proper positioning, ensuring elbow, wrist and fingers are held straight, and fingertips resting on a tripod platform. The team member holds an iPad with a 3D camera attached, and keeping the camera focused on the limb while moving in a specific sequence scans the front, top and back of the arm (Figure 2). The right arm is scanned followed by the left, as per the manufacturer’s specifications. After both arms are scanned, the LymphaTech software calculates the volume for each limb (ml) and shows 3D images of the scanned arms on the iPad screen. Once measurements are obtained, the relative volume change (RVC) to weight-adjusted volume change (WAC) ratio is determined and entered into the REDCap entry linked to the appropriate patient encounter for the designated pre-/postoperative visit.

Our protocol defines clinical BCRL as ≥10% RVC [25] or WAC [28] after treatment for unilateral or bilateral breast cancer, respectively. Subclinical lymphedema is defined as an RVC or WAC of 5–9.9% [29,30]. Measurement data are synchronized across devices at each location, which allows flexibility for patients who may be seen by providers at more than one practice site. Manual backups at the end of each week are conducted to ensure all files are safely stored in the event of technical malfunction.

Although others have shown that symptoms may precede measurable changes in arm volume [31], few existing PSM protocols incorporate PRO assessment into screening. We propose a novel approach to PSM design that utilizes PROs for screening and evaluating program efficacy. This allows the PSM itself to serve as the platform for validating PROMs that prioritize patient-centered outcomes such as financial toxicity [32]. Selection and dissemination of PROMs for our PSM involved consultation with the Patient-Reported Outcomes, Value, and Experience (PROVE) Center at Brigham and Women’s Hospital.

Our protocol uses the Breast Cancer and Lymphedema Symptom Experience Index (BCLE-SEI) to assist with screening [33]. The BCLE-SEI has two subscales: the first, SEI-Symptoms (SEI-S), assesses the degree to which patients may be experiencing symptoms, and the second, SEI-Distress (SEI-D), evaluates how these symptoms may impact QoL.

Patients are asked to complete the SEI-S at baseline so that BCRL-related symptoms that develop post treatment may be distinguished from pre-existing arm morbidity. If one or more symptoms are reported, the patient is prompted to complete the SEI-D subscale. As swelling and heaviness have been shown to be predictive of BCRL [34], the presence of these symptoms in the intervened-upon upper extremity(/ies) triggers a positive screen for potential subclinical/clinical BCRL. Although LymphaTech measurements are simultaneously documented and linked to the survey data for a specific encounter, endorsing swelling and heaviness is sufficient to generate a positive screen regardless of whether volume changes have occurred. The positive SEI-S screen triggers REDCap to generate an automatic email to the lymphedema team coordinators who then contact the patient to offer an appointment for repeat arm measurements and possible intervention.

### 2.6. Early Intervention

**Figure 3:**
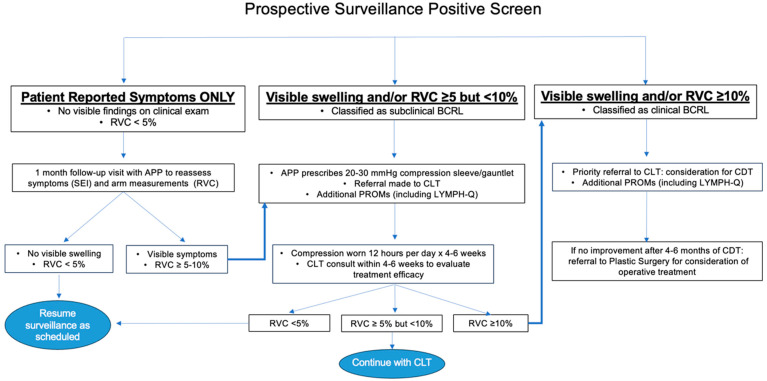
Care pathways for early intervention. Abbreviations: RVC, relative volume change; APP, advanced practice provider; BCRL, breast cancer-related lymphedema; SEI, Symptom Experience Index; CLT, certified lymphedema therapist; PROM, patient-reported outcome metric; CDT, complete decongestive therapy.

Our program specifies a transition from screening into an early intervention care pathway if a patient reports new/worsening limb heaviness or swelling in the absence of significant arm volume changes or if RVC/WAC ≥ 5% (Figure 3).

If a patient reports swelling and/or heaviness but does not have signs of BCRL on clinical exam and RVC/WAC is <5%, a follow-up visit is scheduled with one of our surgical oncology advanced practice providers (APPs) so that symptoms and arm measurements may be re-assessed after a short interval. For ease of workflow and consistency across providers, a templated synoptic note has been designed to ensure consistent documentation (Supplementary Material). The patient will also be seen by a member of the lymphedema team for repeat arm measurements that will be linked to the APP encounter. If no visible signs of BCRL are identified and RVC/WAC remains <5%, the patient reverts back to screening.

If a patient presents with visible swelling and/or RVC/WAC of 5–10% either as the initial finding that prompted a positive BCRL screen or after short-interval follow-up for symptoms alone, an APP or surgical oncologist will prescribe 20–30 mmHg compression garments, a sleeve and gauntlet or glove (depending on the presence of hand swelling), to be worn during waking hours for 12 h per day, removing garments for sleep, for 4–6 weeks [6]. A non-urgent referral to a physical therapist, occupational therapist or CLT is also placed. Ideally, compression is trialed for 4–6 weeks before CLT evaluation so that improvement can be assessed. Following compression, if the patient’s limb volume returns to baseline, they return to the normal surveillance schedule.

An RVC/WAC ≥ 10% at any point during screening or in follow-up after early intervention is initiated prompts a formal diagnosis of clinical BCRL. If this degree of arm volume change represents progression and a CLT has not yet been consulted, a referral is placed for priority scheduling and immediate evaluation. Despite daily use of compression garments, if subsequent patient measurements demonstrate persistence of RVC ≥ 10%, complete decongestive therapy (CDT) is initiated. If RVC remains ≥10% after 4–6 months of CDT and compliance with self-care and daily use of compression garments, a referral for plastic surgery consultation is made. Our colleagues in plastic and reconstructive surgery offer surgical management options to address lymphatic flow disruptions and chronic changes due to BCRL, including lymphovenous bypass, vascularized lymph node transplant and liposuction.

As a regional and national referral center, many of the patients seen at DFBCC live at a distance that precludes frequent follow-up at our main campus. If a patient’s CLT is not within our system, our PSM team establishes contact with the patient’s local CLT and assists in care coordination by providing them with baseline and postoperative volume measurements. The patient is asked to return to our breast surgery group for updated measurements 4–6 weeks after initiating treatment, when it may be more convenient, to assess efficacy of early compression and determine need for continued compression. Returning to our institution for repeat arm measurements using the LymphaTech device is important for an accurate understanding of changes in symptoms and volume over time.

In order to assess the efficacy of the early intervention program, in addition to the SEI-S and SEI-D, patients are invited to complete additional PROMs focused on evaluating the program’s impact on patient-centered outcomes. The LYMPH-Q Upper Extremity Module (LYMPH-Q) [35] comprises six subscales focused on arm function, appearance, symptoms, psychological well-being and satisfaction with BCRL information and treatment. Additionally, as our group has demonstrated the impact of arm morbidity on long-term financial difficulty [36,37], patients are asked about financial hardship as measured by the COmprehensive Score for financial Toxicity (COST) [38].

Prospective screening and early intervention was implemented as part of clinical care for breast cancer patients at high risk for developing treatment-associated lymphedema in January 2023. Retrospective data were used to inform incremental improvements in clinical care; data were abstracted from the Dana-Farber Brigham Cancer Center institutional Clinical Outcomes and Quality Database (COQD), a prospectively maintained database that contains detailed disease and treatment data for 14,636 patients with stage 0-III breast cancer (Dana-Farber Cancer Institute IRB 17-620). Written informed consent will be obtained prior to collecting PROM and interview-based data that will be collected as part of implementation studies to evaluate clinical care (Dana-Farber Cancer Institute IRB 24-196).

## 3. Analysis

Analysis of our PSM and early intervention pathway will involve understanding feasibility of pathway components (i.e., follow-up appointments and serial survey administration), program efficacy and validity of applying relevant PROMs within the context of screening for subclinical and clinical BCRL.

### 3.1. Assessing Feasibility, Organizational Readiness and Implementation

We intend to examine feasibility from both the patient and provider perspectives. We will assess the percentage of enrolled patients who complete all PSM activities (appointments and surveys) over a 2-year span. Characteristics of participants versus non-participants in our population of eligible patients at high risk for developing BCRL will be compared.

As indicated previously, existing implementation studies highlight the importance of process recognition and approval by healthcare providers and staff [10]. In order to facilitate stakeholder engagement, the PSM team presents programmatic updates at a monthly breast oncology provider meeting. Facility staff, advanced practice providers and representatives from multidisciplinary oncology are encouraged to provide feedback on PSM procedures. These occur in coordination with monthly meetings between the PSM team and leadership of the Division of Breast Surgery to discuss how best to resolve concerns and optimize workflow. Meeting minutes are recorded and stored on the Division’s Microsoft SharePoint content management platform.

In addition to eliciting staff feedback at regularly scheduled meetings, our clinical team seeks to formally investigate facilitators and barriers to feasibility and implementation from both the provider and patient perspectives. Providers involved in the PSM (surgeons, physician’s assistants, certified lymphedema therapists [CLTs]) and their patients will be recruited by an experienced member of the Survey and Qualitative Methods Core in the Division of Population Sciences at the Dana-Farber Cancer Institute to participate in semi-structured interviews eliciting feedback using questions inspired by the Reach, Effectiveness, Adoption, Implementation, and Maintenance (RE-AIM) framework [36]. In addition, the Qualitative Methods Core will assist in designing and performing interviews, and coding data using thematic analysis [39].

### 3.2. Evaluating Program Efficacy

Efficacy of the screening program will be evaluated both by assessing whether incorporating PROMs into screening increases BCRL diagnosis and by evaluating whether referral to early intervention improves PROMs and arm measurements. BCRL and subclinical BCRL rates will be recorded for those with at least 2 years of surveillance data after surgery. Demographic, disease and treatment characteristics of these patients will be used to identify a matched cohort of patients using data from the Dana-Farber COQD who were treated in the five years prior to PSM development. Rates of BCRL for patients in the PSM will be compared to these historical controls. To evaluate the efficacy of early intervention, pre- and post-intervention subscale scores and arm measurements will be compared at 6 months and 1 year after subclinical or clinical BCRL diagnosis.

### 3.3. Validating Relevant PROMs

The PSM has been created not only as a clinical intervention for reducing the incidence and severity of BCRL but also as an academic initiative that provides the infrastructure for psychometric development and validation. With respect to the latter, initial endeavors have been aimed at identifying the following: (1) the best PROM for symptom screening, (2) whether selected PROMs are relevant to those with subclinical as well as clinical BCRL and (3) validation of PROMs that assess economic sequelae of BCRL that may impact long-term QoL and cancer treatment adherence. Ultimately, having a single PROM that can serve as a tool for BCRL screening and outcome assessment will reduce survey fatigue and simplify implementation.

To understand whether the LYMPH-Q may be used in the same capacity as the SEI, initial phases of the PSM ask participants to respond to both SEI and LYMPH-Q scales for the purposes of criterion validity testing [40]. LYMPH-Q, like other PROMs used in BCRL, is validated only in populations with clinical BCRL (i.e., RVC ≥ 10%). Our group takes advantage of the PSM to establish the legitimacy of LYMPH-Q scales among those with subclinical BCRL (i.e., RVC 5–9.99%) so that a single instrument might be used for both groups of patients, and changes in these domains can be tracked if a patient’s symptoms improve or progress. One long-term consequence of BCRL that we postulate can be particularly detrimental to QoL is unemployment/disability. Presently, we are field testing additional LYMPH-Q subscales germane to assessing the role of arm-related morbidity and BCRL in ability to return to work. As loss of productivity and employment may impact financial well-being, our group is interested in understanding whether the PSM may be a viable solution for reducing financial toxicity by mitigating a treatment-related adverse event that is known to compromise earning potential and be a source of out-of-pocket expense.

### 3.4. Progress to Date

**Figure 4:**
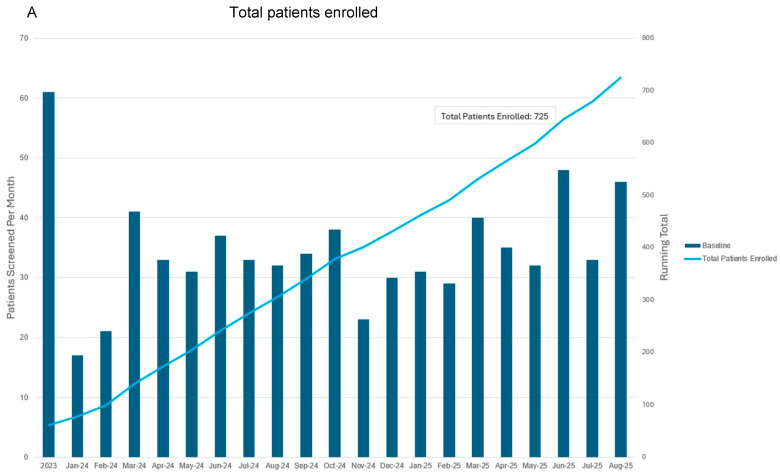

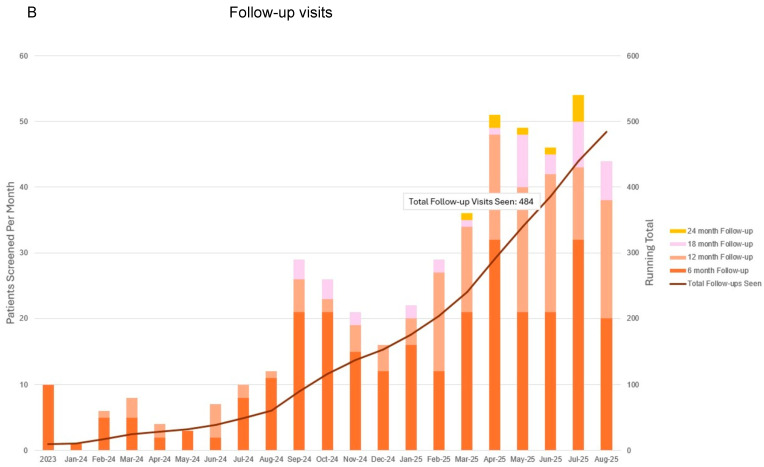
Total patients enrolled (**A**) and follow-up visits to date (**B**) since commencing prospective surveillance and early intervention program. This representation includes follow-up for all patients including those that entered into the program prior to when patient-reported outcome metrics were integrated in March 2024. Bars correspond to patients screened per month as indicated with the vertical axis on the left. Blue and orange lines correspond to total number of patients screened and in follow-up as indicated by the right vertical axis.

The first iteration of this screening program began in January 2023 (Figure 4). PROs were integrated into the screening program in March 2024. Follow-up is ongoing for all patients, including those that enrolled prior to PRO integration. After 2 years of follow-up postoperatively, patients are followed annually. Nine patients have screened positive for subclinical lymphedema and six have screened positive for BCRL. Table 2 describes demographic and clinical characteristics for all patients who had received BCRL screening at the Dana-Farber Brigham Cancer Center between January 2023 and October 2024 when interim analysis was performed.

## 4. Concluding Remarks: Future Directions

The development and implementation of a prospective lymphedema screening program represents a crucial step forward in the comprehensive care of breast cancer survivors. By focusing on early detection and intervention, our initiative aims to mitigate the physical, social and psychological impacts of BCRL. Through the utilization of validated measurement tools such as the LymphaTech device and PRO surveys, this program not only provides personalized care but also contributes to the advancement of medical knowledge and evidence-based practices. Looking ahead, the continued expansion of this screening program holds great promise in further enhancing the QoL for our breast cancer survivors. In particular, our interest in understanding how symptoms and function affect return to work and job performance will provide much-needed data on interventions to safeguard against vocational disruption from BCRL-associated disability. Additionally, iterative analysis of data from individuals enrolled in the screening program, combined with a retrospective chart review of patients who develop BCRL but were not initially enrolled, will support ongoing refinement of eligibility criteria by identifying which other risk factors warrant consideration. Finally, our pioneering efforts to incorporate financial toxicity screening into management of treatment-related adverse events represents, to our knowledge, the first attempt at establishing a need for financial navigation during survivorship. These data will lay the foundation for additional care pathways to provide supportive services. Through collaboration and innovation between various disciplines and members of our provider team, we will continue to refine and strengthen our approach to lymphedema screening and management, ultimately improving outcomes and QoL for our patients with breast cancer.

## Figures and Tables

**Table 1:** Screening criteria for potential eligibility for BCRL screening.

Criteria for Enrollment	Details
Tumor size > 5 cm	Any receptor status
Lymph node involvement	Any receptor status
HER2 + disease	Tumor size > 2 cm
ER/PR-HER2-	Tumor size > 1.5–2 cm

Abbreviations: BCRL, breast cancer-related lymphedema; ER, estrogen receptor; HER2, human epidermal growth factor receptor 2; PR, progesterone receptor.

**Table 2:** Demographic and clinical characteristics of patients enrolled in the Dana-Farber Brigham Cancer Center’s prospective surveillance and early intervention program since January 2023.

Characteristic	*n*/N (%) *n* = 348
Age at diagnosis (years)	
≤40	93 (26.7%)
40–65	186 (53.4%)
≥65	69 (19.8%)
BMI	
<30	244 (70.1%)
≥30	104 (29.9%)
Race	
White	266 (76.4%)
Black	37 (10.6%)
Asian/Pacific Islander	25 (7.2%)
American Indian/Aleutian	1 (3%)
Other	2 (0.6%)
Unknown	17 (4.9%)
Ethnicity	
Not Hispanic/Latino	318 (91.4%)
Hispanic/Latino	22 (6.3%)
Unknown	8 (2.3%)
Sex at birth	
Female	341 (98.0%)
Male	7 (2.0%)
Handedness	
Left	21 (6.0%)
Right	268 (77.0%)
Unknown	59 (17.0%)
Laterality of breast cancer	
Right	171 (49.1%)
Left	165 (47.4%)
Bilateral	12 (3.4%)
Clinical T stage	
T0	3 (0.9%)
T1	77 (22.1%)
T2	189 (54.3%)
T3	50 (14.4%)
T4	26 (7.5%)
Clinical N stage	
N0	126 (36.2%)
N1	187 (53.7%)
N2/3	33 (9.5%)
Nx	2 (0.6%)
Treatment	
Neoadjuvant chemotherapy	324 (93.1%)
Neoadjuvant endocrine therapy	7 (2.0%)
Upfront surgery	15 (4.3%)
Other	2 (0.6%)
Type of surgery	
Partial mastectomy	110 (31.6%)
Total mastectomy with reconstruction	76 (21.8%)
Total mastectomy without reconstruction	65 (18.7%)
Other	5 (1.4%)
Surgery not yet completed	92 (26.4%)
Axillary staging	
Sentinel lymph node biopsy	119 (34.2%)
Axillary lymph node dissection	134 (38.5%)
None	3 (0.9%)
Surgery not yet completed	92 (26.4%)
Prophylactic lymphovenous bypass	4 (1.2%)
Radiation regimen	
None	28 (8.0%)
WBRT with RNI	44 (12.6%)
WBRT without RNI	36 (10.3%)
PMRT with RNI	69 (19.8%)
PMRT without RNI	11 (3.2%)
Radiation not yet completed	160 (46.0%)

Abbreviations: BMI: Body Mass Index; WBRT: Whole Breast Radiation Therapy; RNI: Regional Nodal Irradiation; PMRT: Post-Mastectomy Radiation Therapy.

## Data Availability

De-identified data from this study are not available in a public archive. De-identified data from this study will be made available (as allowable according to institutional IRB standards) by emailing the corresponding author.

## Supplementary Materials

The following supporting information can be downloaded at https://www.mdpi.com/article/10.3390/jcm14197051/s1.
