# Supplementary material for: Preliminary Efficacy/Feasibility Study of a Breast Cancer-Related Lymphedema Prospective Screening and Early Intervention Program at the Dana-Farber Brigham Cancer Center
Source: J Clin Med. 2025 Oct 6;14(19):7051. doi: 10.3390/jcm14197051 (PMC12524797; doi:10.3390/jcm14197051)
Supplement: Supplementary file 1 [file jcm-14-07051-s001.zip › jcm-3838015-supplementary.pdf]

Subclinical Lymphedema

When you started your treatment for breast cancer you were enrolled in our Lymphedema Screening Program and initial measurements were taken of both arms. More recent measurements of your arms reveal that there have been changes in your affected arm, demonstrating that you have **subclinical lymphedema.**

What is Subclinical Lymphedema?

Subclinical Lymphedema is a slight buildup of lymph fluid in the affected arm. Swelling or symptoms may not yet be apparent to you or easily detectable by your clinical team; however, changes were detected when we compared your recent arm measurements to your baseline measurements.

Subclinical lymphedema is the earliest stage of arm swelling, also referred to as Stage 0 lymphedema. Not all patients with subclinical lymphedema will progress to more advanced stages of lymphedema, yet there is good evidence that **early intervention** can reverse these changes and prevent progression.

What is Early Intervention?

Early intervention includes wearing a compression garment daily for a limited period of time, and consulting with a certified lymphedema therapist. Recent studies have demonstrated that wearing a compression garment for 12hrs per day for 4 weeks, sometimes longer, can reduce swelling and prevent the progression to lymphedema. It is important to wear the garment while you are awake and active, you should not wear the compression garments during sleep. Consultation with a certified lymphedema therapist will include recommendations for future use of compression garments, and additional risk reducing strategies.

What happens next?

1) Referral for a Compression Sleeve and Gauntlet:

- Your clinical team will provide you with prescriptions for a compression sleeve (for your arm) and a compression gauntlet (for your hand), The strength of the compression for both will be 20-30mmHg. https://www.lymphedemaproducts.com/products/juzo-compression-sleeves.html

- If convenient, you can contact Dana Farber Friends Place to obtain your compression garments. You will need to schedule an appointment for fitting. Inform them you are a patient in the Breast Surgery Lymphedema Screening Program.
Ph: 617-632-2211

- Alternatively, contact a local Durable Medical Supplier closer to your home and inquire if they do fittings for compression arm sleeves and gauntlets.

- Garments should feel somewhat tight, but supportive and comfortable to wear all day. Garments should not cause pain or numbness and tingling in arm or hand. They should be washed every 1-2 days to maximize their effectiveness.

- Wear garments for 12hrs per day while awake, remove for sleeping. Continue to wear garments until you see a certified lymphedema therapist, ideally 4-6wks from now.

2) **Referral to a Certified Lymphedema Therapist:**
   - Please call to schedule an appointment with a certified lymphedema therapist at Brigham and Women's Faulkner Hospital (BWFH). Ideally this appointment would take place 4 to 6 weeks after you start wearing the compression garments, but please do not wait to call to schedule:
     Ph: 617-983-7271. Address: 1153 Centre St. Boston, MA 02130

   - If BWFH is not convenient for you, please check this website for a certified lymphedema therapist near you: http://clt-lana.org. Schedule an appointment for 4 to 6wks after obtaining your compression garments.

How will I know this is effective?

When you meet with a therapist you will have repeat arm measurements. You will know if early intervention with compression garments is effective if there is no progression of swelling in your affected arm. If your arm measurements return to normal you will not require additional treatment and we will continue to perform arm measurements at your regular follow-up visits with your breast cancer team. If you notice any changes between these visits, please contact us sooner for new measurements.

What happens if swelling increases in my arm?

If repeat measurements show that lymphatic fluid build-up is progressing in your affected arm your certified lymphedema therapist will recommend additional treatments to help reduce and manage arm swelling.

Any questions?

If you have any questions about these recommendations, fit of compression garments, or change in symptoms please contact us at **bwhbreastsurgeryle@bwh.harvard.edu**

Lymphedema Fact Sheet

What is lymphedema?

Lymphedema is the accumulation of lymphatic fluid that can cause swelling in the arm and/or hand. Lymphatic fluid is normally filtered through the lymph nodes. Removal of lymph nodes requires lymph fluids from the arm to be rerouted and filtered through remaining axillary lymph nodes. Lymphedema occurs in a small number of patients, and symptoms can range from hand swelling alone to total arm swelling. Should you notice any swelling, please contact your surgeon's office who will instruct you in appropriate follow up care. Intervention includes physical or occupational therapy, manual lymphatic drainage, compression bandaging and garments. New research suggests gradual, progressive strengthening, when cleared by your physician, can actually minimize the risk of lymphedema by dilating, or widening, remaining lymphatic channels around the shoulder and arm.

Who is at risk for lymphedema?

With a sentinel node biopsy, the lifetime risk of lymphedema is very small and may occur in up to 5% of patients. With an axillary dissection or radiation to the axilla, the lifetime risk is up to 20%. The vast majority of cases of lymphedema related to breast surgery or radiation occur in the first year after treatment. Being overweight can increase your risk.

What are the early signs of lymphedema?

The early sign of lymphedema is swelling of the arm or hand. Sometimes the arm will feel heavy. Sometimes the first sign is that your sleeve, watch or jewelry feels tighter than usual.

Can I prevent lymphedema?

While there is no medical evidence that lymphedema can be prevented, below are some recommendations for possible risk reduction:

- Maintaining a healthy weight has been shown to reduce risk of lymphedema.

- Try to avoid cuts and infections in your affected arm
    - Be careful with sharp objects or edges.
    - Wear gloves while gardening or performing housework.
    - Protect yourself from insect bites.
    - Avoid sunburns by using sunscreen with SPF 30 or higher.
    - Wash all cuts with soap and water.
    - Regularly use a moisturizer to avoid skin cracking.

- Blood pressure readings, blood injections, tests and infusions
    - There is no evidence that these will cause lymphedema. If needed, it is safe to have these performed in either arm. However, your preferences on usage/selection of arm for these tests should be respected.
    - Research shows that you should avoid blood draws and blood pressure in the arm <u>only</u> if you have been diagnosed with lymphedema or have history of lymphedema in that arm.

- Exercise
    - There is strong evidence that exercise does not cause lymphedema and does not worsen lymphedema for patients who have been diagnosed with it.
    - When beginning a strengthening program or new exercise routine, start with light weights or a short time period and increase weight and time period gradually. Monitor your arm for any changes.

- Airplane travel
    - If you have not been diagnosed with lymphedema, there is no evidence that you need to wear a compression sleeve while traveling for prevention.
    - Stretch your arm often during and before your flight.

- Saunas and Hot tubs
    - There is no medical literature that shows hot tubs cause lymphedema.
    - One study suggested that saunas may play a role in the development of lymphedema. If you would like to use a sauna, start with gradual exposure and monitor your body for swelling.

Breast Edema

A relatively common complication of breast conserving treatment (lumpectomy with or without radiation) is breast edema. This happens when normal inflammatory fluid from surgery and/or radiation gets "trapped" in the superficial breast tissue, specifically just under the skin. It is more common in patients with larger breast sizes and after receiving radiation. This can cause the breast to appear swollen, heavy, achy, and sometimes painful. Due to gravity, the fluid settles at the bottom of the breast and the skin may appear thickened and firm. Symptoms often improve with the following treatments, but in rare cases it can become a longstanding issue that requires continued management.

Treatments:

- Regular exercise and use of an extra supportive bra and NSAIDS (Advil, ibuprofen, or Aleve) to help with symptoms. Ice packs may help with comfort as needed.

- Physical therapy or occupational therapy referral for evaluation, lymphatic massage techniques, and if necessary, compression garment fitting (bra, tank tops, etc.) if painful or bothersome

There are physical and occupational therapists who specialize in lymphedema care. Your medical provider will give you a referral if needed. You may also have a pre-operative meeting with a physical therapist if you are interested in learning more about lymphedema care.

Questions?

If you have any questions or would like more information about lymphedema treatment and prevention, please email us at bwhbreastsurgle@bwh.harvard.edu

Supplement 2: Synoptic note to facilitate consistent documentation

CC: Follow-up LE screening
HPI: @NAME@ is a @AGE@ {Gender:76141} with history of {Laterality:76181} breast cancer s/p {Previous Treatment for Breast Cancer:76136} who presents for follow-up after a positive screen for {Symptoms/RVC changes:76176}.
Today, {Pronouns:76210} reports {Free Text:76177} .
PE:
@VITALS@

Bilateral Upper Extremities
Pain:{Pain Affected Arm:76137}
Signs of infection: {Signs of Infection:76139}
Presence of visible edema: {Presence of visible edema:76178}
RVC measurement performed today?: {RVC measurement:76177}

Assessment/plan: @Name@ is a @AGE@ {Gender:76141} presenting today for LE screening follow-up.
Based on today's assessment, patient will {Lymphedema Plan:76180}.
